# E2F1 and TFDP1 Regulate *PITX1* Expression in Normal and Osteoarthritic Articular Chondrocytes

**DOI:** 10.1371/journal.pone.0165951

**Published:** 2016-11-01

**Authors:** Martin Pellicelli, Cynthia Picard, DaShen Wang, Patrick Lavigne, Alain Moreau

**Affiliations:** 1 Viscogliosi Laboratory in Molecular Genetics of Musculoskeletal Diseases, Sainte-Justine University Hospital Research Center, Montréal, Québec, Canada; 2 Department of Biochemistry and Molecular Medicine, Faculty of Medicine, Université de Montréal, Montréal, Québec, Canada; 3 Orthopedic Division, Maisonneuve-Rosemont Hospital, Montréal, Québec, Canada and Department of Surgery, Faculty of Medicine, Université de Montréal, Montréal, Québec, Canada; 4 Department of Stomatology, Faculty of Dentistry, Université de Montréal, Montréal, Québec, Canada; Università degli Studi di Milano, ITALY

## Abstract

We previously reported a loss-of-PITX1 expression in patients suffering of knee/hip osteoarthritis (OA). Search for the mechanism underlying this event led us to discover that PITX1 repression was triggered by the aberrant nuclear accumulation of Prohibitin (PHB1), an E2F1 co-repressor, in OA articular chondrocytes. In the current study, we assessed in details the involvement of E2F transcription factors in regulating *PITX1* expression. We also analyzed other genes that are similarly regulated by E2F in regard to osteoarthritis. The transcriptional regulation of the *PITX1* promoter by E2F1 was analyzed with the luciferase reporter assay, and chromatin immunoprecipitation assays, which confirmed direct E2F1-*PITX1* interactions. The probable binding sites for E2F1 in the *PITX1* promoter were identified by DNA pulldown experiments. *In silico* and *in vitro* analyses show that the *PITX1* proximal promoter region contains 2 specific sequences that are bound by E2F1. Overexpression of E2F1 enhances *PITX1* promoter activity and mRNA transcription. In primary control and osteoarthritis chondrocytes, real time RT-PCR was used to measure the mRNA expression levels of candidate genes under E2F1 transcriptional control. Transcription Factor Dp-1 (TFDP1) knockdown experiments confirmed that the E2F1-TFDP1 complex regulates *PITX1*. Knockdown of *TFDP1*, an E2F1 dimerization partner, inhibits the activating effect of E2F1 and reduces both *PITX1* promoter activity and mRNA transcription. Real time RT-PCR results reveal reduced expression of *TFDP1* and a similar downregulation of their targets *PITX1*, *BRCA1*, *CDKN1A*, and *RAD51* in mid-stage OA chondrocytes. Collectively, our data define a previously uncharacterized role for E2F1 and TFDP1 in the transcriptional regulation of *PITX1* in articular chondrocytes. Additional E2F1 targets may be affected in OA pathogenesis.

## Introduction

The progression of osteoarthritis (OA) is characterized by an initial biosynthetic phase followed by a degradation phase, where tissue damage becomes irreversible [1]. To improve our understanding of cartilage and bone homeostasis in the context of OA, we previously studied a mouse model developing early OA symptoms at the age of 7 months [2]. Compared with age-matched control mice, *Pitx1*+/- mice showed bone thickening and articular cartilage calcification. The complete knockout of the *Pitx1* gene resulted in severe reduction of knee cartilage elements [3]. The proximal ends of *Pitx1*-/- mice tibias at E17.5 were devoid of articular cartilage due to the absence of secondary ossification centers. The expanded layer of hypertrophied chondrocytes and the decreased mineralization around those cells suggest impaired apoptosis [4]. Indeed, impaired chondrocyte apoptosis during the process of endochondral ossification would prevent blood vessel invasion, resulting in the absence of secondary ossification centers [5].

In human cartilage samples, we previously showed a general downregulation of *PITX1* (MIM# 602149; NP_002644) mRNA and protein levels when OA patients were compared with control patients [2]. We recently demonstrated that the downregulation of *PITX1* is due to the nuclear accumulation of Prohibitin 1 [6]. To further understand how *PITX1* regulation could be associated with the progression of osteoarthritis, we investigated potential positive regulators. Microarray expression analyses showed that E2F1 (MIM# 189971; AAH58902), E2F2 (MIM# 600426; AAH53676.1), and E2F3 (MIM# 600427; AAN17846.1) upregulated *PITX1* expression levels by several folds in the human osteoblast cell line U2OS [7]. The proteins E2F1-3 belong to the E2F transcription factor family and are known as the activating E2Fs as opposed to E2F4-8 that are mainly considered repressors [8]. All activating and repressing E2Fs (except E2F7-8) need to dimerize with TFDP1 (Transcription Factor Dp-1; MIM# 189902; NP_009042.1) or TFDP2 (MIM# 602160; AAH21113.1), to bind DNA. The E2F family members are differentially associated with other factors and can activate distinct sets of target genes. The E2F target genes regulate cell cycle regulation (e.g. cyclins A and E or CDKN1A), nucleotide synthesis (e.g. thymidine kinase), DNA repair (e.g. BRCA1 and RAD51), and many other functions [9].

While E2F members participate mainly in cell proliferation and differentiation, E2F1 is the only member specifically involved in apoptosis [8]. The E2F1 transcription factor was proposed to act as a specific signal for the induction of apoptosis by mediating the accumulation of the p53 protein (TP53) [10]. Interestingly, the *TP53* gene has been shown to be directly regulated by the transcription factor PITX1 [11]. These findings prompted us to further investigate if E2F1 could directly regulate *PITX1* gene expression levels. Results from the present study reveal, for the first time, that *PITX1* is a direct target of E2F1, and other targets may also be regulated in a similar manner during the progression of osteoarthritis. To clarify the emerging roles of E2F transcription factors and their targets in OA pathogenesis, we will discuss their functions in cartilage and bone homeostasis.

## Materials and Methods

### Human cartilage samples

Four control cartilage specimens were obtained from the knees of deceased or trauma patients without osteoarthritis (2 females and 2 males, mean age 44 ± 28 years). Osteoarthritis (OA) cartilage specimens were obtained from the tibial plateaus and femoral condyles of 18 OA patients (12 females and 6 males, mean age 65 ± 21 years) undergoing total knee joint replacement. A certified rheumatologist evaluated all the OA patients based on the American College of Rheumatology Subcommittee diagnostic guidelines. Prior to surgery, all the patients underwent radiological assessment and were evaluated based on the Kellgren-Lawrence grading scale: KL 0; 1 –control (none / doubtful); KL 2 / 3 –mid-stage OA (minimal / moderate); KL 4 –end-stage OA (severe) [12]. Human tissues were collected with the written consent of the patients or of their relatives in the case of postmortem tissues. The institutional review boards of Sainte-Justine University Hospital and Maisonneuve-Rosemont Hospital approved the study protocol.

### Primary chondrocyte isolation and cell culture

Unless specified, all the reagents were obtained from Sigma-Aldrich Co. LLC. (MO, USA) and BioShop Canada Inc. (ON, Canada). For primary chondrocyte extraction and culture, the normal and OA human cartilage were sectioned, rinsed, and finely minced. They were digested first with 0.25% trypsin for 1 hour at 37°C, rinsed with phosphate-buffered saline (PBS) and then digested with 2 mg/ml collagenase for 4 to 6 hours at 37°C. The cells were seeded in Falcon culture flasks at high density (10^8^ cells per 175-cm^2^ flask) and grown in Dulbecco’s Modified Eagle’s Medium (Thermo Fisher Scientific Inc., MA, USA) containing 10% heat-inactivated fetal calf serum (GE Healthcare Life Sciences, UT, USA) and 1% penicillin and streptomycin. For gene expression analyses, the subconfluent cells were harvested after the first passage.

The human C28/I2 chondrocyte cell line was kindly provided by Dr. Mary Goldring, (Hospital for Special Surgery, New York, NY, USA) and cultured, as previously described [13]. To generate stable C28/I2 cells overexpressing E2F1, a retroviral infection using PHOENIX cells (kind gift obtained from Dr. Gerardo Ferbeyre, Université de Montréal) was performed, as previously described [14].

### RNA extraction and real time RT-PCR analysis

RNA extraction, cDNA synthesis, and real-time RT-PCR analysis were performed, as previously described [6]. Primer sequences are listed in Table 1.

**Table 1:** List of primers used in mRNA detection by real-time RT-PCR.

Gene symbol	GeneID	Orientation	Nuclotide sequence
*ACTB*	60	Forward	5'_GGAAATCGTGCGTGACAT_3'
		Reverse	5'_TCATGATGGAGTTGAAGGTAGTT_3'
*BRCA1*	672	Forward	5'_AGGAAATGGCTGAACTAGAAG_3'
		Reverse	5'_TCTGGATTCTGGCTTATAGGG_3'
*CDKN1A*	1026	Forward	5’_GTCACTGTCTTGTACCCTTG_3’
		Reverse	5’_GGCGTTTGGAGTGGTAG_3’
*E2F1*	1869	Forward	5'_GTCCAAGAACCACATCCAGTG_3'
		Reverse	5'_TCCTGGGTCAACCCCTCAAG_3'
*E2F2*	1870	Forward	5'_TGGGTAGGCAGGGGAATGTTTG_3'
		Reverse	5'_AGTCACATAGGCCAGCCTCTTG_3'
*E2F3*	1871	Forward	5'_AAGCGGTCATCAGTACCTCTCAG_3'
		Reverse	5'_CAAGAGACGTATCATACCGCGTT_3'
*PITX1*	5307	Forward	5'_GCTACCCCGACATGAGCA_3'
		Reverse	5'_GTTACGCTCGCGCTTACG_3'
*RAD51*	5888	Forward	5’_ATGAGTTTGGTGTAGCAGTG_3’
		Reverse	5’_CAGGGAGAGTCGTAGATTTTG_3’
*TFDP1*	7027	Forward	5'_ACGTCTAACGGCACAAGGTT_3'
		Reverse	5'_CTGAGACCCATTGGAGCTTG_3'

### Vector constructs

To clone the human *PITX1* promoter region, the -3895/+61 bp region was first amplified using genomic DNA extracted from the MG-63 osteoblast cell line (ATCC, VA, USA). Specific primer pairs (sequences available upon request) containing the KpnI and EcoRV restriction sites were used for subsequent cloning of the different DNA regions in the pGL4 vector.

### Luciferase assay

Luciferase assays were performed in C28/I2 cells, as previously described [6].

### Chromatin immunoprecipitation (ChIP)

Chromatin immunoprecipitation (ChIP) using primary chondrocytes or C28/I2 cells and anti-E2F1 antibodies was performed, as previously described [6].

### Nuclear extract and DNA pulldown

Five 10 cm-dishes of confluent C28/I2 cells were washed 3 times and scraped using cold PBS. The cells were centrifuged at 500 x g for 5 minutes at 4°C. They were then resuspended in 2 ml buffer A (10 mM HEPES pH 7.9, 1.5 mM MgCl_2_, 10 mM KCl, 1% NP-40, 500 nM DTT, and 1X Complete EDTA-free protease inhibitor cocktail—Hoffman-La-Roche, ON, Canada) and incubated for 25 minutes on ice with short vortexing pulses every 3 minutes, to allow cytoplasm disruption. This homogenate was centrifuged at 600 x g for 10 minutes at 4°C to pellet the crude nuclear fraction. This fraction was resuspended in buffer B (50 mM Tris-HCl pH 7.6, 2 mM EDTA, 2 mM EGTA, 1 mM DTT, 0.1% TritonX-100, and 1X Complete EDTA-free protease inhibitor cocktail), layered over 30% sucrose (in buffer B without TritonX-100), and centrifuged at 3500 x g for 60 minutes at 4°C. The pellet containing the pure nuclear fraction was resuspended in buffer C (10 mM Tris-HCl pH 7.6, 100 mM KCl, 1 mM EDTA, 500 nM DTT, 5% glycerol, 0.4% NP-40, and 1X Complete EDTA-free protease inhibitor cocktail) and sonicated on ice for three 15-second pulses with an output of 7, using the Sonifier S-150D from Branson Ultrasonics Corporation (CT, USA). The nuclear extract was centrifuged at 13 000 x g for 10 minutes at 4°C to pellet the debris, and the protein concentration was determined with a protein assay kit from Bio-Rad Laboratories Ltd. (QC, Canada).

For the DNA pulldown, 250 μg of the nuclear extract underwent pre-clearing using 30 μl neutravidin agarose beads (Thermo Fisher Scientific Inc.) with incubation for 2 hours at 4°C on a rocker-type shaker. The pre-cleared nuclear extracts were then incubated in 500 μl buffer C containing poly dAdT nonspecific oligonuclotides (4 ng/μl) and specific double-stranded biotinylated probes (4 ng/μl) (Table 2) and placed on the shaker for 16 hours at 4°C. For competition, 1X (4 ng/μl) or 3X (12 ng/μl) specific double-stranded non-biotinylated probes were added to the mix. For double-stranded biotinylated probes, each antisense oligonucleotide was biotinylated at its 5′ end to allow interaction with neutravidin. All the oligonucleotides were obtained from Integrated DNA Technologies, Inc. (IA, USA). The DNA/protein complexes were captured using 50 μl of neutravidin agarose beads for 1.5 hours at 4°C on a rocker-type shaker. After incubation, the beads were washed 4 times with buffer C and resuspended in 3X Laemmli loading buffer. The proteins were resolved by SDS-PAGE gel, transferred to a PVDF membrane, and immunodetected with specific antibodies.

**Table 2:** List of probes used in the DNA pulldown assay.

Probe name and PITX1 promoter region	Foward (f)or Reverse (r)	Probe sequence
-195 (-195/-165 bp)	f	5'-CGAGGCCGCGGGGGGCGGGGAGGCGGCGGG-3'
	r	5'-(biotin)CCCGCCGCCTCCCCGCCCCCCGCGGCCTCG-3'
-180 (-180/-150 bp)	f	5'-CGGGGAGGCGGCGGGAAGGTGGCTGCGGAG-3'
	r	5'-(biotin)CTCCGCAGCCACCTTCCCGCCGCCTCCCCG-3'
-170 (-170/-140 bp)	f	5'- GCGGGAAGGTGGCTGCGGAGGGGGAGGGCG-3'
	r	5'-(biotin)CGCCCTCCCCCTCCGCAGCCACCTTCCCGC-3'
-115 (-115-85 bp)	f	5'-GCGGCAGTGAGGGCGCGGCGGCGCGGGCGG-3'
	r	5'-(biotin)CCGCCCGCGCCGCCGCGCCCTCACTGCCGC-3'
-100 (-100/-70 bp)	f	5'-CGGCGGCGCGGGCGGCTTGGGGCTGGATTC-3'
	r	5'-(biotin)GAATCCAGCCCCAAGCCGCCCGCGCCGCCG-3'
-180m1 (-180/-150 bp)	f	5'-ATTTGAGGCGGCGGGAAGGTGGCTGCGGAG-3'
	r	5'-(biotin)CTCCGCAGCCACCTTCCCGCCGCCTCAAAT-3'
-180m2 (-180/-150 bp)	f	5'-CGGGGATTATGCGGGAAGGTGGCTGCGGAG-3'
	r	5'-(biotin)CTCCGCAGCCACCTTCCCGCATAATCCCCG-3'
-180m3 (-180/-150 bp)	f	5'-CGGGGAGGCGGATTTAAGGTGGCTGCGGAG-3'
	r	5'-(biotin)CTCCGCAGCCACCTTAAATCCGCCTCCCCG-3'
-115m4 (-115/-85 bp)	f	5'-TATTCAGTGAGGGCGCGGCGGCGCGGGCGG-3'
	r	5'-(biotin)CCGCCCGCGCCGCCGCGCCCTCACTGAATA-3'
-115m5 (-115/-85 bp)	f	5'-GCGGCAGTGAGTTATCGGCGGCGCGGGCGG-3'
	r	5'-(biotin)CCGCCCGCGCCGCCGATAACTCACTGCCGC-3'
-115m6 (-115/-85 bp)	f	5'-GCGGCAGTGAGGTATAGGCGGCGCGGGCGG-3'
	r	5'-(biotin)CCGCCCGCGCCGCCTATACCTCACTGCCGC-3'
-115m7 (-115/-85 bp)	f	5'-GCGGCAGTGAGGGATATGCGGCGCGGGCGG-3'
	r	5'-(biotin)CCGCCCGCGCCGCATATCCCTCACTGCCGC-3'

### Gene knockdown using siRNA

To silence the TFDP1 expression, the cells were transfected with 300 pmol of Validated Stealth RNAi siRNA (Thermo Fisher Scientific Inc.) and 15 μl of Lipofectamine RNAiMAX Transfection Reagent (Thermo Fisher Scientific Inc.), according to the manufacturer’s instructions. The TFDP1 siRNA specifically recognized the sequence GCAGACGAGCUGGUUGCGGAGUUCA. After 48 or 72 hours, the cells were lysed in RIPA buffer for protein analysis or TRIzol Reagent (Thermo Fisher Scientific Inc.) for mRNA analysis.

### Antibodies

For Western blots or chromatin immunoprecipitation assay, mouse monoclonal anti-E2F1 (KH95; sc-251x; AB_627476; 1:2000), rabbit polyclonal anti-E2F2 (L-20; sc-632x; AB_2277708; 1:2000), rabbit polyclonal anti-E2F3 (C-18; sc-878x; AB_2096807; 1:2000), and rabbit polyclonal anti-TFDP1 (K-20; sc-610x; AB_2202574; 1:2000) antibodies were purchased from Santa Cruz Biotechnology Inc. (TX, USA). Rabbit polyclonal anti-PITX1 (ab70273; AB_1269775; 1:5000), mouse monoclonal anti-Lamin A/C (4777S; AB_10545756; 1:2000), and mouse monoclonal anti-Tubulin (B-5-1-2; AB_792484; 1:10 000) antibodies were purchased from Abcam plc. (MA, USA), Cell Signaling Technology, Inc. (MA, USA), and Sigma-Aldrich Co. LLC. (MO, USA), respectively.

## Results

### Basal *PITX1* promoter activity levels are elevated following the overexpression of E2F1, E2F2, or E2F3

**Fig 1:**
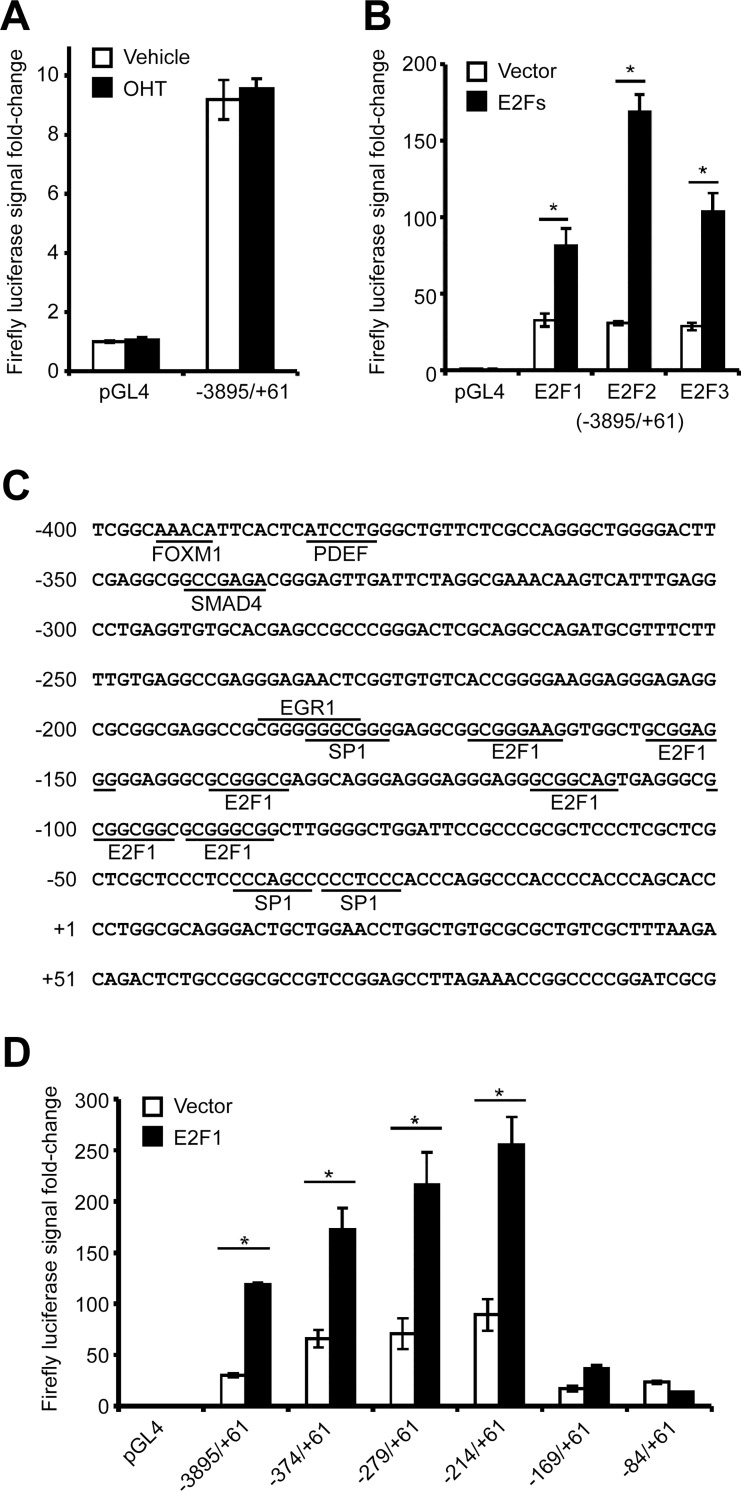
Critical regulatory regions in the *PITX1* promoter govern its expression in chondrocytes.

In order to identify the key regulatory regions of the human *PITX1* promoter, a long fragment of DNA corresponding to the -3895/+61 *PITX1* gene region was cloned in a luciferase reporter vector (pGL4). The level of activity for this region, represented by the firefly luciferase signal intensity, reached approximately 9-fold when compared with the empty pGL4 vector (Fig 1A). Treatment with 4OH-tamoxifen (OHT), which was used to activate the different pBabe-ER-E2F constructs, did not affect the intensity of the signal.

To quantify the effect of E2F1, E2F2, and E2F3 on the -3895/+61 *PITX1* gene region, the C28/I2 human chondrocytes were co-transfected with tamoxifen-inducible forms of E2F. The overexpression of functional E2F1, E2F2, or E2F3 was sufficient to significantly activate the -3895/+61 *PITX1* gene region (Fig 1B). Similar to Müller's results, all 3 E2Fs were able to activate the *PITX1* gene with E2F2 providing the strongest signal [7]. However, *in silico* analyses of the *PITX1* promoter region, using MatInspector 8.0 software from Genomatix Software Suite [15], indicated several proximal binding sites more specifically used by E2F1 (Fig 1C). Furthermore, E2F1 is the only E2F family member able to bind to PHB1, the main focus of our previous study (6). These reasons prompted us to further investigate the effect of E2F1 on the *PITX1* proximal promoter.

Smaller fragments of the *PITX1* promoter region (-374/+61, -279/+61, -214/+61, -169/+61 and -84/+61) were cloned into the pGL4 vector. Following their transfection in the C28/I2 cell line, the effect of E2F1 overexpression on the promoter activity of the smaller fragments was analyzed (Fig 1D). The results showed that E2F1 overexpression produced a significantly increased luciferase signal with most promoter regions, except the -169/+61 and the -84/+61 constructs. The highest fold-change in luciferase activity, which reached approximately 250-fold, was observed with the -214/+61 construct. The dramatic loss of signal obtained with the -169/+61 construct suggested the proximity of critical regulatory elements.

Human C28/I2 chondrocyte cells were cotransfected with either the empty pGL4 plasmid (luciferase reporter plasmid) or the pGL4 plasmids containing different regions of the *PITX1* promoter combined with either the empty pBabe plasmid or pBabe plasmids expressing ER (estrogen receptor) fused to E2F1, E2F2, or E2F3 and induced with 4OH-tamoxifen (OHT) for 24 h. (**A)** The -3895/+61 *PITX1* gene region contains regulatory elements capable of producing a luciferase signal that is not affected by OHT treatment. (**B)** Overexpression of E2F1, E2F2, and E2F3 produces a significant increase in the luciferase activity under the control of the -3895/+61 *PITX1* gene region. (**C)** The proximal sequence of the *PITX1* promoter contains several E2F1 binding sites, as predicted by MatInspector 8.0 software (Genomatix Software Suite). (**D)** Overexpression of E2F1 has variable effects on luciferase activity depending on the length of the transfected promoter region (-3895/+61; -374/+61; -279/+61; -214/+61; -169/+61; -84/+61). Except for the -84/+61 *PITX1* gene region, all the other constructs are significantly activated by E2F1. **(Fig 1A, 1B and 1D**) Data represents mean and standard deviation of 3 independent experiments. Asterisks represent a significant increase in luciferase activity (Two-way ANOVA; Bonferroni *post hoc*: *p < 0.0001) compared with control cells.

### E2F1 upregulates *PITX1* expression levels by directly binding to its proximal promoter region

**Fig 2:**
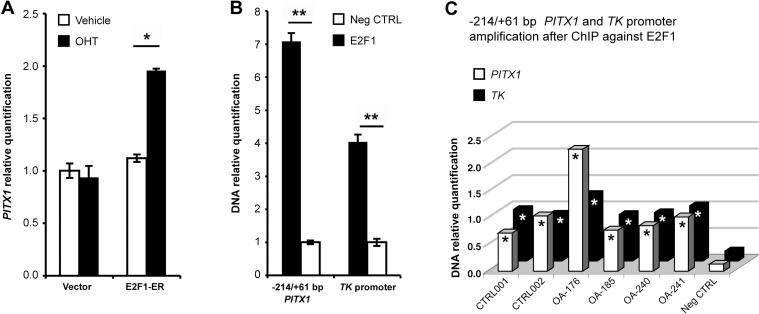
E2F1 directly regulates *PITX1* gene expression in the C28/I2 cell line and in primary chondrocytes. **(A)** Real time RT-PCR performed on the *PITX1* gene in C28/I2 chondrocytes stably expressing pBabe-ER-E2F1 fusion plasmids or the empty control vector, treated with OHT or vehicle (100% ethanol) 24 hours prior to RNA extraction. Data represent mean ± standard deviation of 3 independent experiments. Asterisks represent a significant increase in OHT-treated cells compared with vehicle-treated cells (Two-way ANOVA; Bonferroni *post hoc*: *p < 0.0001). (**2B-2C)** Chromatin immunoprecipitation (ChIP) assays of E2F1 on *PITX1* and *TK* promoters in (**B)** C28/I2 chondrocytes and (**C)** primary chondrocytes. Data are presented as relative quantification of DNA following immunoprecipitation when compared with the negative control. Error bars represent standard deviation of 3 independent experiments (Two-way ANOVA; Bonferroni *post hoc*: *p < 0.05, **p < 0.0001).

To validate if E2F1 has an effect on the *PITX1* promoter of endogenous origin, we overexpressed E2F1 followed by RNA extraction. Stable expression of E2F1 in C28/I2 chondrocytes resulted in a significant upregulation (approximately 2-fold) of *PITX1* gene expression levels (Fig 2A).

To test if this effect was direct, ChIP assays were performed using anti-E2F1 antibodies and specific primers amplifying the -214/+61 *PITX1* proximal promoter region. A nonspecific antibody was used as a negative control, and the thymidine kinase (TK) promoter region, containing known E2F1 binding sites, was used as a positive control. In C28/I2 cells, the results showed that E2F1 was recruited to the *PITX1* proximal promoter as well as the *TK* promoter (Fig 2B).

To verify if E2F1 binding was affected by osteoarthritis disease, the same ChIP experiments were performed in control chondrocytes as well as in OA chondrocytes. Results showed that E2F1 was recruited to *PITX1* and *TK* promoters in a similar manner in all the chondrocytes tested except for OA patient 176 (Fig 2C).

### E2F1 binds to two specific response elements in the *PITX1* proximal promoter region

**Fig 3:**
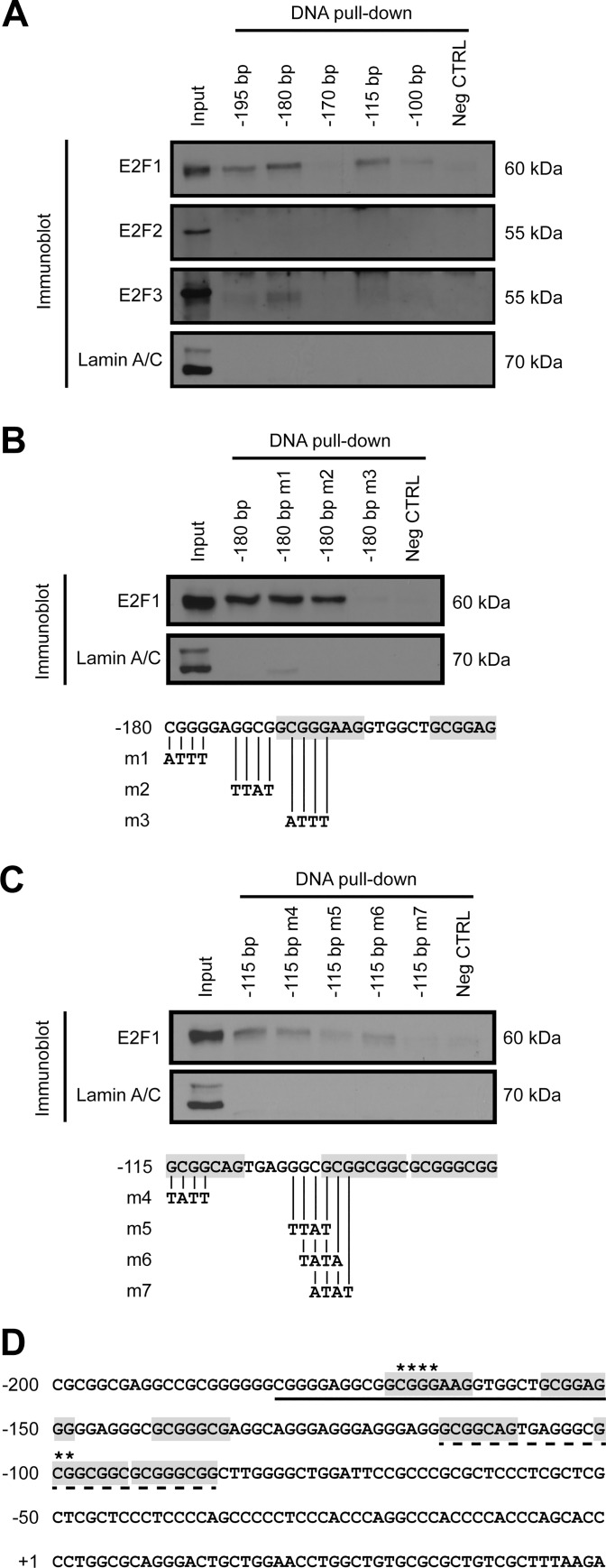
Point mutation analyses in the C28/I2 chondrocyte *PITX1* promoter confirm specific E2F1 binding sites. Biotinylated double-stranded probes representing different wild type or mutated regions of the *PITX1* promoter were used in DNA pulldown assays. A nonspecific 30 bp biotinylated double-stranded probe was used as a negative control. In the representative immunoblots, lamin A/C was used as a loading control for the nuclear extract. (**A**) Different 30 bp probes corresponding to distinct *PITX1* proximal promoter regions were used as baits to detect specific binding with E2F1, E2F2, or E2F3. (**B**) Fine mapping of E2F1 binding the -180 bp probe: m1, m2, m3 (point mutations). (**C**) Fine mapping of E2F1 binding the -115 bp probe: m4, m5, m6, m7 (point mutations). (**D)** Sequence of the -225/+75 bp *PITX1* promoter region according to the transcription start site (+1). Predicted E2F1 response elements by MatInspector 8.0 software from the Genomatix Software Suite are highlighted in grey. The -180 bp and -115 bp probes are underlined with a dark and a dashed line, respectively. Asterisks represent the nucleotides that are essential for the binding of E2F1 to the *PITX1* proximal promoter.

To identify precisely which response element was bound by E2F1 to activate the *PITX1* promoter, DNA pulldown assays were performed with different probes followed by immunoblotting using specific antibodies. Five different double-stranded biotinylated probes corresponding to -195/-165, -180/-150, -170/-140, -115/-85, and -100/-70 *PITX1* promoter regions were incubated with nuclear extracts from the C28/I2 cells (Fig 3A). Immunoblots against E2F1, E2F2, and E2F3 were performed to verify binding specificity. The use of anti-E2F2 antibodies did not give any positive results, while E2F3 interacted weakly with the -180/-150 probe. Interestingly, E2F1 interacted with all the probes except with the -170/-140 probe. The strongest interactions were found between E2F1 and the -180/-150 probe followed by the -115/-85 and -195/-165 probes.

Since several E2F1 response elements are present in the -195/-70 *PITX1* gene region, we performed more DNA pulldown assays using probes with point mutations in order to identify specifically which response element is bound by E2F1 to activate the *PITX1* promoter. We first mutated 3 regions of the probe -180/-150, referred to as m1, m2, and m3 (Fig 3B). We observed that mutation m3 completely abolished the interaction between E2F1 and the probe but mutations m1 and m2 did not affect the E2F1/DNA interaction. We thus identified 5'-GCGGGAAG-3' as the first response element bound by E2F1 to activate the *PITX1* promoter.

We then mutated four regions of the probe -115/-85, referred to as m4, m5, m6, and m7 (Fig 3C). We noticed that only mutation m7 abrogated the interaction between E2F1 and the probe. This result indicated that E2F1 uses a second response element to activate the *PITX1* promoter, corresponding to the predicted sequence 5'-GCGGCGGC-3'. Consistent with the luciferase assay experiments (Fig 1D), these results confirmed that E2F1 activates *PITX1* promoter by interacting with at least two response elements, with the first response element producing a stronger activation than the second one (see asterisks on Fig 3D).

### TFDP1 knockdown reduces *PITX1* expression levels and interferes with the activating effect of E2F1

**Fig 4:**
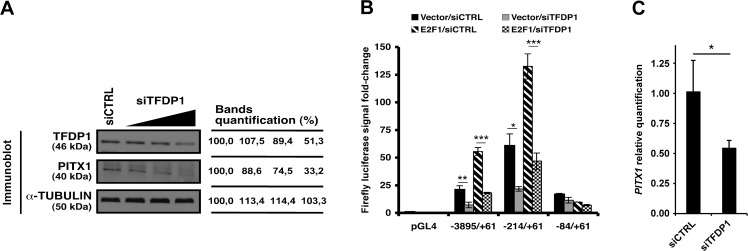
TFDP1 knockdown affects *PITX1* gene regulation. **(A)** Immunoblots of TFDP1 and PITX1 were performed in C28/I2 cells transfected with a nonspecific siRNA (siCTRL) or increasing amounts of TFDP1 siRNA (siTFDP1). α-TUBULIN was used as a loading control for immunoblotting. The percentage values of the relative expression of TFDP1 and PITX1 are indicated. **(B)** Luciferase assays were performed in C28/I2 cells transiently transfected with different constructs of the *PITX1* promoter. The cells were co-transfected with either the pBabe plasmid expressing ER fused to E2F1 or the empty control vector along with either control siRNA or TFDP1 siRNA, and were induced with 4OH-tamoxifen (OHT) for 24 hours. The data represent mean ± standard deviation of 3 independent experiments. Asterisks represent a significant decrease in the luciferase activity (Two-way ANOVA; Bonferroni *post hoc*: *p < 0.01, **p < 0.001, ***p < 0.0001) compared with control cells. **(C)** Real time RT-PCR analysis of *PITX1* mRNA in chondrocytes from healthy subjects transfected with either control siRNA or TFDP1 siRNA. Data represent mean ± standard deviation of 3 independent experiments. Asterisks represent a significant decrease in *PITX1* levels in siTFDP1-transfected cells compared with control (Student's t-test: *p < 0.02).

To test the involvement of TFDP1 in *PITX1* gene regulation, *TFDP1* knockdown was performed using specific siRNA. Functional knockdown of *TFDP1* in the C28/I2 chondrocytes resulted in reduced PITX1 protein levels (Fig 4A). The *TFDP1* siRNA produced a dose-dependent response with 33% of PITX1 protein levels corresponding to a 51% reduction in TFDP1 expression. This could be explained by the fact that DP1 can also interact with p53 and activate transcriptional targets (including *PITX1*) by binding to E2F sites [16].

To verify if TFDP1 knockdown could inhibit the activating effect of E2F1, luciferase experiments were performed with selected regions of the *PITX1* promoter. The TFDP1 depletion resulted in a significant decrease in the luciferase signal for both the -3895/+61 and the -214/+61 constructs (Fig 4B). When siTFDP1 was transfected while E2F1 was overexpressed, the activating effect of E2F1 was significantly diminished using the -3895/+61 bp promoter fragment and the -214/+61 region, but not using the -84/+61 region. These results suggest that TFDP1 likely uses the same binding sites as those previously identified for E2F1.

We then tested the effect of TFDP1 depletion in control primary chondrocytes. Results show that the transfection of TFDP1 siRNA induced a 50% reduction in *PITX1* mRNA levels (Fig 4C). These results suggest that TFDP1 and E2F1 could jointly regulate *PITX1* expression levels in OA pathogenesis.

### E2F1, TFDP1, and specific targets are differentially expressed in primary chondrocytes based on OA severity

**Fig 5:**
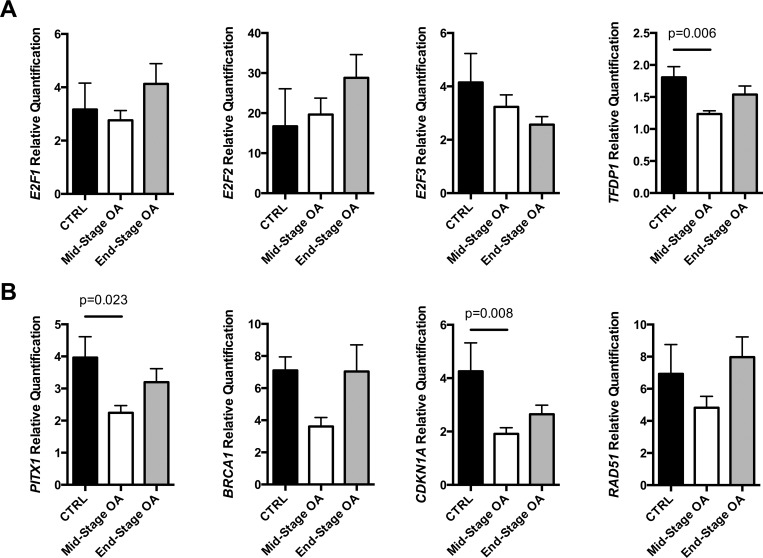
Gene expression levels of E2F family members and selected targets in primary articular chondrocytes. RNA was extracted from primary cultures of chondrocytes derived from the knees of 4 controls and 18 OA patients. The control subjects (CTRL) were attributed Kellgren-Lawrence (KL) scores of 0 (n = 3) and 1 (n = 1) while OA patients accounted for KL scores of 2 (n = 6), 3 (n = 5), and 4 (n = 7). Since chondrocyte proliferation may influence E2F-related gene expression levels, samples with KL 2–3 scores (mid-stage OA characterized with increased proliferation) were separated from KL 4 (end-stage OA). **(A)** Real time RT-PCR analysis of the E2F family members *E2F1*, *E2F2*, *E2F3*, and *TFDP1*. **(B)** Real time RT-PCR analysis of the E2F targets *PITX1*, *BRCA1*, *CDKN1A*, and *RAD51*. (**A-B**) Y-axis: relative expression (fold changes) compared with the lowest score of 1. (One-way ANOVA followed by Bonferroni *post hoc*: only significant p < 0.05 indicated on graphs).

To determine if activating E2F family members or their dimerization partner TFDP1 were differentially regulated during the onset or progression of OA, real time RT-PCR experiments were performed. *E2F2* and *E2F3* showed no significant changes in their expression levels among the patients tested (Fig 5A). Interestingly, *TFDP1* levels varied among OA patients, stratified by severity using the radiological Kellgren-Lawrence (KL) score. *TFDP1* is significantly downregulated in articular chondrocytes obtained from mid-stage OA patients (KL 2–3) compared with control patients (KL 0–1). However, an upregulation of *TFDP1* was observed in cells derived from end-stage OA patients with a higher radiological score (KL 4) when compared with the previous ones. Of the 3 E2Fs found in partnership with TFDP1, only E2F1 showed a similar pattern of expression.

To determine which E2F targets could be affected during the course of OA, additional real time RT-PCR experiments were performed on the same set of patients. The expression levels of *PITX1*, *BRCA1* (MIM# 113705), *CDKN1A* (MIM# 116899), and *RAD51* (MIM# 179617) (Fig 5B) revealed a pattern similar to that of *TFDP1* (Fig 5A), with *PITX1* and *CDKN1A* being statistically significant. Other known E2F targets like *CCNA2* and *CCNE1* were not affected (data not shown).

## Discussion

Our data show that E2F1 significantly upregulates *PITX1* expression levels in human chondrocytes and interacts directly with the *PITX1* promoter through the following proximal E2F1 binding sites: -170 5'-GCGGGAAG-3' -163 and -101 5'-GCGGCGGC-3' -94. The first E2F1 response element seemed more important in activating *PITX1*, as demonstrated by our luciferase and DNA pulldown experiments. While most E2F members share similar DNA binding properties, both *in silico* and *in vitro* point mutation analyses indicate that E2F1 is the main transcription factor directly involved in *PITX1* regulation (Fig 3A). Experiments showing that E2F2 and E2F3 are able to activate the *PITX1* promoter may indicate an indirect effect, mediated by other transcription factors, or by using other binding sites. The overexpression of E2F2 and E2F3 could also provoke a positive effect by sequestering important transcriptional repressors.

Since the main heterodimeric partner for E2F1 is TFDP1 [17], we wanted to verify the contribution of TFDP1 in *PITX1* gene regulation. The knockdown of TFDP1 in human chondrocytes downregulated PITX1 at the mRNA and protein levels. Luciferase assays confirmed that E2F1 activity was reduced by low TFDP1 levels. Interestingly, *TFDP1* mRNA levels were differentially expressed in primary chondrocytes, based on OA severity. The progression of OA was first marked by *TFDP1* downregulation followed by upregulation in the advanced stage. Several E2F targets, including *PITX1*, showed a similar pattern of expression during OA progression. TFDP1 can dimerize with most E2Fs to act as a transcriptional regulator, but results from this study suggest that E2F1 could be the main member involved in OA pathogenesis.

In the literature, the E2F transcription factors have been studied extensively, however, not in the context of osteoarthritis. Our results suggest that many E2F targets could be affected during the progression of OA. The *CDKN1A* gene (cyclin-dependent kinase inhibitor 1A, also known as p21 or the wild-type p53-activated fragment 1) was previously shown to be downregulated in OA cartilage cells compared with normal cells [18]. Due to its ability to block proliferation in many cell types, the loss of *CDKN1A* was proposed to mediate the re-initiation of cell proliferation in the OA cartilage. Our experimental data suggest that this phenomenon could be more pronounced in OA patients with KL scores of 2 and 3 (mid-stage OA). In patients with KL scores of 0, 1, and 4, higher levels of *CDKN1A* could suggest a diminished proliferation rate. Indeed, the overexpression of *CDKN1A* in OA synovial fibroblasts was shown to induce a G0/G1 cell cycle arrest, without inducing cytotoxicity [19].

Cell cycle arrest is a protective mechanism that allows the effective repair of damaged DNA. Increased levels of *CDKN1A* correlate with *RAD51* expression and foci formation, which are linked with recombinational DNA repair [20]. The multiple steps of damage signaling also involve BRCA1, well known for its involvement in breast and ovarian cancers [21]. Of note, BRCA1 was shown to inhibit cell growth by tethering UBC9, resulting in enhanced nuclear localization [22]. In a recent study, BRCA1 was identified among the top 10 transcription factors differentially expressed and contributing in the development of OA [23]. No direct links have been established between RAD51 and osteoarthritis pathogenesis. However, a significant increase in DNA damage is observed in OA chondrocytes [24]. Significant DNA damage can induce apoptosis, which is known to correlate with OA severity [25]. Thus, in patients with a KL score of 4, elevated levels of known E2F targets like *CDKN1A*, *RAD51*, and *BRCA1*, could likely be associated with DNA damage and cell death.

The emerging roles of E2F1/TFDP1 and PITX1 in cartilage homeostasis and OA progression, highlighted in this study, will open up new avenues for future investigations.
