# Correction: E2F1 and TFDP1 Regulate *PITX1* Expression in Normal and Osteoarthritic Articular Chondrocytes

**DOI:** 10.1371/journal.pone.0167530

**Published:** 2016-11-23

**Authors:** The PLOS ONE Staff

The caption for Fig 1 is incorrectly displayed as the fourth paragraph of the Results section. The publisher apologizes for the error. Please see the correct Fig 1 caption here.

**Fig 1:**
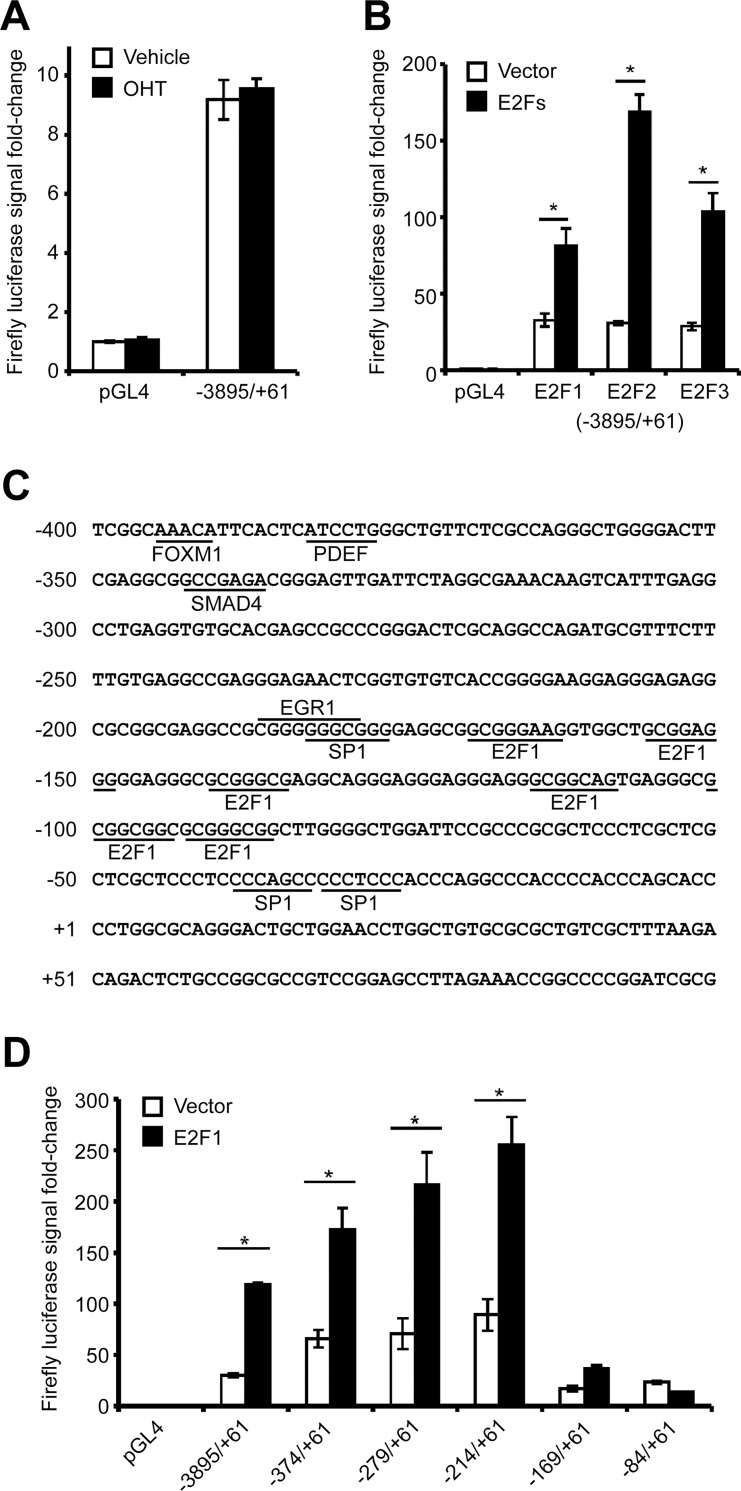
Critical regulatory regions in the *PITX1* promoter govern its expression in chondrocytes. Human C28/I2 chondrocyte cells were cotransfected with either the empty pGL4 plasmid (luciferase reporter plasmid) or the pGL4 plasmids containing different regions of the *PITX1* promoter combined with either the empty pBabe plasmid or pBabe plasmids expressing ER (estrogen receptor) fused to E2F1, E2F2, or E2F3 and induced with 4OH-tamoxifen (OHT) for 24 h. **(A)** The -3895/+61 *PITX1* gene region contains regulatory elements capable of producing a luciferase signal that is not affected by OHT treatment. **(B)** Overexpression of E2F1, E2F2, and E2F3 produces a significant increase in the luciferase activity under the control of the -3895/+61 *PITX1* gene region. **(C)** The proximal sequence of the *PITX1* promoter contains several E2F1 binding sites, as predicted by MatInspector 8.0 software (Genomatix Software Suite). **(D)** Overexpression of E2F1 has variable effects on luciferase activity depending on the length of the transfected promoter region (-3895/+61; -374/+61; -279/+61; -214/+61; -169/+61; -84/+61). Except for the -84/+61 *PITX1* gene region, all the other constructs are significantly activated by E2F1. **(Fig 1A, 1B and 1D)** Data represents mean and standard deviation of 3 independent experiments. Asterisks represent a significant increase in luciferase activity (Two-way ANOVA; Bonferroni *post hoc*: *p < 0.0001) compared with control cells.
